# Mycological air contamination level and biodiversity of airborne fungi isolated from the zoological garden air — preliminary research

**DOI:** 10.1007/s11356-024-33926-2

**Published:** 2024-06-18

**Authors:** Kinga Plewa-Tutaj, Paweł Krzyściak, Aleksandra Dobrzycka

**Affiliations:** 1 Department of Microbial Ecology and Acaroentomology, Faculty of Biological Sciences, University of Wroclaw, Wrocław, 51-148 Poland; 2 Department of Infection Control and Mycology, Chair of Microbiology, Jagiellonian University Medical College, Czysta 18, 31-121 Kraków, Poland

**Keywords:** Fungi, Microbial air contamination, Molecular identification, *Aspergillus*, *Penicillium*, Zoological garden

## Abstract

The aim of this paper was to evaluate the degree of mycological air contamination and determine the taxonomic diversity of airborne fungi residing in the air of 20 different animal facilities in a zoological garden. The concentrations of fungi in the zoological garden were measured using a MAS-100 air sampler. The collected microorganisms were identified using the combination of molecular and morphological methods. The fungal concentration ranged from 50 to 3.65 × 10^4^ CFU/m^3^ during the whole study. The quantitative analysis of the fungal aerosol showed that the obtained concentration values were lower than the recommended permissible limits (5 × 10^4^ CFU/m^3^ for fungi). Environmental factors, including temperature and relative humidity, exerted a varying effect on the presence and concentration of isolated fungi. Relative humidity was shown to correlate positively with the concentration of fungal spores in the air of the facilities studied (rho = 0.57, *p* < 0.0021). In parallel, no significant correlation was established between temperature and total fungal concentration (rho =  − 0.1, *p* < 0.2263). A total of 112 fungal strains belonging to 50 species and 10 genera were isolated. *Penicillium* was the dominant genera, including 58.9% of total fungal strains, followed by *Aspergillus* 25.89%, *Cladosporium* 3.57%, *Talaromyces* 3.57%, *Mucor* 1.78%, *Schizophyllum* 1.78%, *Syncephalastrum* 0.89%, *Alternaria* 0.89%, *Absidia* 0.89%, and *Cunninghamella* 0.89%. Our preliminary studies provide basic information about the fungal concentrations, as well as their biodiversity in zoological garden. Further studies are needed to generate additional data from long-term sampling in order to increase our understanding of airborne fungal composition in the zoological garden.

**Supplementary Information:**

The online version contains supplementary material available at 10.1007/s11356-024-33926-2.

## Introduction

Zoological gardens are one of the most popular attractions visited by tourists worldwide. We should perceive them not only as places where a large diversity of animals is kept, but also as places where people can admire both native and exotic species (Nekolný and Fialová 2018). Both visitors and zoo workers are potentially exposed to bioaerosols which contain bacteria, viruses, pollens, fungi, and mycotoxins (Michalska et al. 2021; Nageen et al. 2023). Among the microorganisms in bioaerosols, fungi are the most numerous group of biological particles (Szulc et al. 2020). Those microorganisms are abundant in the environment and play important roles as symbionts, saprotrophs, or parasites. It is estimated that airborne fungi constitute nearly 25% of the global biomass and, therefore, play a significant role in air pollution affecting human health (almost 150 fungal taxa are associated with allergies) (Nageen et al. 2023).

National and global research about the contamination and the biological diversity of fungi in breeding facilities was carried out mainly in the large-scale poultry houses, barns, and piggeries (Plewa and Lonc 2011; Pusz et al. 2015; Douglas et al. 2018; Seifi et al. 2018; Lee and Kim 2021). Against this background, research on fungal biodiversity and the degree of air and environmental pollution in zoological gardens are scarce (Rivas et al. 2018; Cateau et al. 2022; Álvarez-Pérez et al. 2023; Debergh et al. 2023). The majority of these cited works primarily concentrate on fungi of the genus *Aspergillus*, with a particular focus on *Aspergillus fumigatus*, its sensitivity to azole drugs, and the detection of fungi solely in the environment of a single animal group (penguins). They do not usually consider the presence of fungi in other animal habitats and another group of molds. Moreover, the only national study on microbial air contamination was carried out in Krakow’s Zoo (Grzyb and Lenart-Boroń 2019, 2020). The research focused entirely on the determining occurrence of individual bioaerosol (bacterial and fungal) fractions, without taking into account the biodiversity of fungi and their potential toxicity. The studies conducted in animal breeding places other than zoos often showed high concentrations of this group of microorganisms that can be harmful to human and animal health. Among the isolated fungi, the most frequently identified were fungi belonging to the genera *Aspergillus* (*A. fumigatus*, *A. niger*, *A. flavus*), *Penicillium* (*P. citrinum*, *P. viridicatum*), *Cladosporium* spp., *Alternaria* spp., and *Scopulariopsis* spp. (Plewa and Lonc 2011; Pusz et al. 2015; Seifi et al. 2018). The presence of these poses a risk of disease in people with bronchial asthma, EAA (extrinsic allergic alveolitis), allergic rhinitis, and ODTS (dust-induced toxic syndrome organic), while in animals, it causes pulmonary aspergillosis and mycotoxicosis (Szulc et al. 2020).

Therefore, research in zoological gardens is important because these facilities are not only places for breeding animals but may be a source of dangerous fungi. This is important information, taking into account the specificity of zoos, which are not only a working environment, but also one of the most frequently visited tourist places. Thus, the aim of this paper was to evaluate the degree of mycological air contamination and determine the taxonomic diversity of airborne fungi residing in the air of different animal facilities using the combination of microscopic and genetic analyses.

## Materials and methods

### Study area

The study was conducted at the Zoological Garden in Wroclaw. This zoo has the largest collection of animals in Poland, with almost 1100 different species in an area covering 33 hectares (https://zoo.wroclaw.pl).

The measurements were carried out inside twenty facilities: two sites of Monkey House, four sites of Apes Pavilion (*Pan troglodytes*), Papio Pavilion (*Papio anubis*), three cages with maggots* (Macaca sylvanus)*, five sites in Kongo Pavilion (with crocodiles, manatees, and numerous species of birds), and five sites in East Africa (with the *Hippopotamus amphibious*, *Orycteropus afer*, *Heterocephalus glaber*). The choice of study sites was guided by their convenient accessibility.

### Sampling methods

Air samples were taken in October 2022 and in January, April, and June 2023 using a MAS-100 air sampler (Merck KgaA, Darmstadt, Germany). Three parallel samples (two incubated at 27 °C degree, one at 37 °C) were collected at the center point of each location at a height of 1.5 m above the ground and directly struck on the surface of Sabouraud agar. The plates were incubated for about 7 days at 25 °C (two samples) and 37 °C (one sample). The number of fungal colonies was expressed as a total colony-forming units (CFU/m^3^) and revised by the equation: Pr = N[1/N + 1/N − 1 + 1/N − 2 + 1/N − r + 1] where Pr, *r*, and *N* stand for revised colonies, number of viable colonies, and the number of sieve pores, respectively (Feller 1968). The concentration of airborne microorganisms (CFU/m^3^) was calculated according to the following formula: *X* = (*a* × 1000)/*V*, where “*a*” is the number of fungal colonies, and “*V*” is the air volume sampled (m^3^) (Michalska et al. 2021).

## Characterization of meteorological conditions

The air temperature and relative humidity were measured during each sampling session using a thermo-hygrometer (HI9565 HANNA, Poland). The air temperature ranged from 11.2 to 22.7 °C (autumn season), 17.1 to 24 °C (winter season), 18.1 to 24.6 °C (spring season), and 19.4 to 25.6 °C (summer season), respectively. Relative humidity in the autumn season was between 27.9 and 89.4%, in the winter season from 30.8 to 95.5%, in the spring season from 35.5 to 90.8, and in the summer season from 54.7 to 80.1%.

### Identification of fungi

Airborne fungi were identified based on their macro- and microscopic features using diagnostic keys (Samson et al. 2014, 2019; Yilmaz et al. 2014; Visagie et al. 2014). Then, the cultured fungi were subjected to molecular identification to confirm species identity. DNA extraction from the selected and dominant fungi was performed using the Tissue DNA Purification Kit (EURx, Gdańsk, Poland) according to the manufacturer’s instructions. For the diagnostic of the airborne fungi, we used both morphological criteria and molecular analyses based mainly on the sequence of the internal transcribed spacer ITS. In the case of several closely related strains, we sequenced b-tubulin and calmodulin fragment. Amplification parts of the ITS were amplified according to the methods described by White et al. (1990). The ITS was amplified using a pair of primers: ITS1 (5′-TCCGTAGGTGAACCTGCGG-3′) and ITS4 (5′-TCCTCCGCTTATTGATATGC-3′). PCR reactions were performed in a T100 Thermal Cycler (Bio-Rad, Warsaw, Poland) in a total volume of 12.5 µL. Each PCR reaction contains 6.25 μL of 2 × PCR Mix Plus (A&A Biotechnology, Gdansk, Poland), 0.625 μL of each primer (10 mM), 4 μL of DNA template, and 1 μL of ddH_2_O. PCR conditions included an initial denaturation step of 95 °C (30 s); 34 cycles of 95 °C (45 s), 55 °C (60 s), and 72 °C (60 s); and the final elongation of 72 °C (3 min).

Calmodulin gene amplification was performed using the set of primers Cmd5 and Cmd6, and amplification of b-tubulin was performed using the primer pairs Bt2a (5-GGT AAC ATC CAA GCT GCT GGT TTC-3) and Bt2b (5-CTC AGT GTA ACC GTG ACC CTT GGC-3) Glass and Donaldson (1995). For both regions, the conditions were as follows: denaturation step of 95 °C (3 min); 40 cycles of 95 °C (45 s), 55 °C (60 s), and 72 °C (60 s); and the final elongation of 72 °C (7 min). The PCR products were visualized in 1% agarose gel staining with SimplySafe (EURx).

All positive samples obtained in the PCR reaction were purified and sequenced (Macrogen, Amsterdam, the Netherlands) using the same primer pairs as in the PCR reaction. The obtained sequences were manually edited using DNA Baser Sequence Assembly software (Heracle BioSoft SRL Romania), and consensus sequences were aligned and compared with sequences deposited in the National Center for Biotechnology Information’s GenBank (NCBI, Bethesda, MD, USA) using the BLAST algorithm (http://www.ncbi.nlm.nih.gov/). The most representative sequences that form the basis of the phylogenetic tree have been included in the GenBank database (Table 1). Phylogenetic analysis was performed using the Maximum Likelihood method with the MEGA 7.0 software, bootstrapping was performed using 1000 replicates.

**Table 1:** Molecular identification (using ITS region) of fungi isolated from the air in the zoological garden

Fungal species	Isolate	Accession no	Identity
*Aspergillus niger (1)*	7fj	OR807891.1	100%
*Aspergillus niger (2)*	15cj	OR807968.1	100%
*Aspergillus ostianus*	17cj	OR801711.1	100%
*Aspergillus ostianus*	11bl	OR802129.1	100%
*Aspergillus westerdijkiae (1)*	1dz	OR816133.1	99.55%
*Aspergillus westerdijkiae (2)*	4dz	OR816127.1	99.55%
*Aspergillus elegans (1)*	1bz	OR802130.1	99%
*Aspergillus elegans (2)*	2cz	OR802142.1	100%
*Aspergillus steynii*	16cw	OR806947.1	100%
*Aspergillus flavus (1)*	2gw	OR806964.1	100%
*Aspergillus flavus (2)*	18aw	OR806964.1	100%
*Aspergillus fumigatus (1)*	3fw	OR8069661.1	100%
*Aspergillus giganteus*	19cz	OR808008.1	100%
*Aspergillus sydowii*	4bl	OR807981.1	100%
*Aspergillus versicolor*	13cz	OR807986.1	100%
*Penicillium chrysogenum*	12aj	OR810003.1	99.82%
*Penicillium solitum*	15aj	OR810004.1	99,63%
*Penicillium commune*	6dj	OR810005.1	100%
*Penicillium raistrickii*	9bw	OR810006.1	100%
*Penicillium bialowieziense*	9bj	OR810007.1	100%
*Penicillium brevicompactum (1)*	9cj	OR810008.1	100%
*Penicillium olsonii*	13bz	PP494186.1	100%
*Penicillium glandicola*	6aw	OR810010.1	100%
*Penicillium citreosulfuratum*	1az	OR810011.1	100%
*Penicillium glabrum*	8aj	PP494188.1	100%
*Penicillium citrinum (1)*	15az	OR810014.1	100%
*Penicllium citrinum (2)*	15bl	OR810013.1	100%
*Penicilllium steckii*	18cw	OR810015.1	100%
*Penicillium steckii (2)*	20cz	OR810016.1	100%
*Penicillium sumatraense*	15bz	PP494184.1	100%
*Penicillium copticola (1)*	17bz	PP494185.1	100%
*Penicillium copticola (2)*	18az	OR810019.1	100%

### Statistical analysis

The data underwent statistical analysis using R version 4.3.1 in RStudia 2023.09.0. Linear regression models and Spearman correlations were computed to evaluate the correlation between fungal abundance and meteorological parameters (humidity and temperature). The Kruskal–Wallis test and Dunn’s post hoc test for multiple comparisons were utilized to investigate the influence of qualitative factors, such as location and season, on fungal abundance. For statistical significance, results with *p* < 0.05 were considered. Margalef’s Index and Jaccard’s Similarity Index were employed to assess the diversity of fungal species at specific research sites. The Jaccard Index was calculated pairwise, and then the average value for each site was obtained.

## Results and discussion

### Fungal concentrations in air samples

**Fig. 1:**
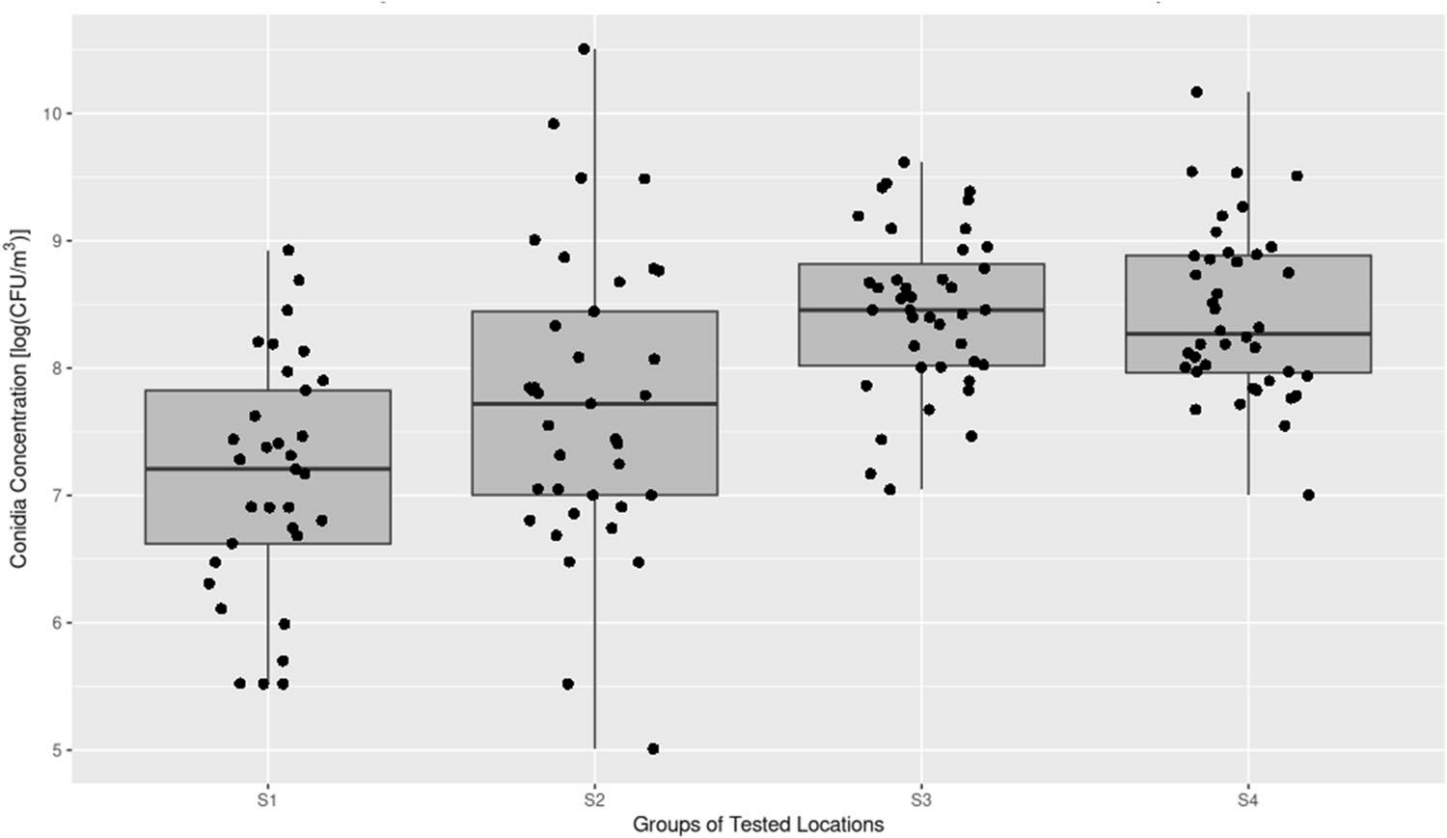
Fungal conidia concentration in the air across locations group

A total of 240 air samples were collected from 20 locations in the zoological garden. Fungi were detected in 234 (97.5%) samples. The concentrations of countable fungal aerosol are presented in Table 2 and ranged from 5.0 × 10^1^ to 3.65 × 10^4^ CFU/m^3^ (for a temperature of 27 °C). The lowest concentration of fungi was recorded in Papio Pavilion 5 × 10^1^ CFU/m^3^ (range 5 × 10^1^ –2.5 × 10^2^ CFU/m^3^). It is worth noticing that this concentration was lower than the recorded at another site, because the air sampling was done just before the morning cleaning of the room. The highest concentration of fungi was recorded in the Apes Pavilion and ranged between 2.02 × 10^4^ and 3.65 × 10^4^ CFU/m^3^. Similar concentrations were found in the Papio Pavilion (range 1.31 × 10^4^–1.32 × 10^2^ CFU/m^3^) and the Kongo Pavilion (1.38 × 10^4^–2.61 × 10^4^ CFU/m^3^). Statistical analysis performed using the Kruskal-Walis test showed statistically significant differences between the sampling location and the CFU/m^3^ fungi values (Fig. 1).

**Table 2:** Air fungal concentration (CFU/m^3^) during seasonal samplings

Fungal count (CFU/m^3^)												
Location	Autumn			Winter			Spring			Summer		
1	250	250	100	750	900	150	1650	2500	750	1000	1350	900
2	300	400	400	250	450	0	550	650	200	850	800	350
3	1500	1300	0	Z	Z	350	2900	3600	350	7500	5950	5500
4	1600	1000	50	Z	Z	1650	3650	4700	150	3400	2700	3900
5	2050	Z	300	Z	Z	800	1000	1700	300	1450	1750	1850
6	20250	36500	150	3250	4650	250	1700	2450	100	650	900	200
7	1400	Z	0	2400	2550	350	1150	1100	100	950	1000	400
8	8150	2550	600	6500	7100	150	1650	800	150	1900	1500	350
9	4150	3200	4000	6400	5850	700	2500	2250	150	850	650	250
10	Z	Z	900	150	250	50	1150	1100	50	13150	13250	250
11	11150	8900	50	5150	5850	1000	12700	15050	1100	2500	3150	2600
12	5950	7550	0	5200	3550	650	9850	7700	3000	3000	2150	850
13	4700	5600	0	2600	3600	250	11950	12300	3200	2700	1750	1650
14	4450	5600	0	3000	4550	600	6500	6000	2900	1150	3050	2100
15	4450	4200	50	5600	4700	1000	8900	4700	3600	1700	1300	250
16	6200	4000	500	3600	4100	750	5350	4950	1750	2500	2800	650
17	13950	7200	350	3500	3600	1700	7700	6850	1500	1900	2350	850
18	13800	26100	400	2400	2900	1350	7000	7400	1450	1100	3350	350
19	13450	3250	250	2550	2250	750	6300	7250	1300	3000	2150	400
20	4750	8700	250	3050	3800	400	9850	10550	1750	2900	2700	450

1–2 (Monkey House), 3–5 (cages with maggots 1), 6–9 (Apes Pavilion), 10 (Papio Pavilion), 11–15 (Kongo Pavilion), 16–20 (East Africa pavilion); Z, overgrown Petri dish

The first two columns in each season concern strains grown at a temperature of 27 °C; the last one is for strains grown at 37 °C

There have been a lot of studies about microbial and mycological air contamination in animal production premises (Plewa and Lonc 2011; Pusz et al. 2015; Matković et al. 2009) or in the farming environment (Radon et al. 2002; Almatawah et al. 2023), but there are only several publications relating to non-production facilities, such as zoos. In Poland, the first study carried out in the zoological garden was initiated by Grzyb and Lenart-Boroń (2019) and focused mainly on bacterial bioaerosol in the selected animal premises in the Krakow’s Zoological Garden. Another study conducted by Grzyb and Lenart-Boroń (2020) inside the premises where animals are kept demonstrated similar “to our work” total concentration of fungi (range 8.4 × 10^2^–2.84 × 10^4^). The highest median concentration of fungi was recorded in the exotarium (range 9.79 × 10^3^–2.84 × 10^4^ CFU/m^3^), and the lowest was recorded in rooms for pygmy hippopotamus 966 CFU/m^3^ (range 8.4 × 10^2^–1.3 × 10^3^ CFU/m^3^) (Grzyb and Lenart-Boroń 2020). Because of the lack of other studies on the mycological quality of air in zoos, the results of our own research can be compared to other breeding facilities, e.g., poultry houses and cowshed. Matković et al. (2007) observed in the barn the average concentration of fungi ranged from 5.23 × 10^4^ CFU/m^3^ (at noon) to 8.35 × 10^4^ CFU/m^3^ (in the morning). The air quality in some farming objects has been described by Radon et al. (2002). According to that author, the total number of fungi in poultry houses and in pig farms was higher than the concentrations of airborne fungi noticed in our research and ranged from 8.3 × 10^4^ to 1.1 × 10^9^ (Radon et al. 2002). Such large differences in the level of microbial contamination between the zoological garden and other breeding premises are most likely due to different methods of air sampling. The sampler used in our investigation has some limitations. This device is characterized by sampling efficiency described by the cuttoff size parameter D50, at the level of 1.7 μm (Górny et al. 2023). All particles above the size would be collected and almost all particles smaller than that size may not be captured by this impactor. Airborne fungal spores typically have an aerodynamic diameter (d_ae_) of 2 to 4 μm, and some species release fragments of 0.3 to 1.3 μm (Madsen et al. 2016). That is why the number of airborne fungi in our research may have been underestimated in relation to the actual concentration in the air. The results of the present study suggest that it is necessary to use the same instruments and methods to assess microbial contamination. The lack of standardization of air sampling methods may lead to incorrect conclusions.

**Fig. 2:**
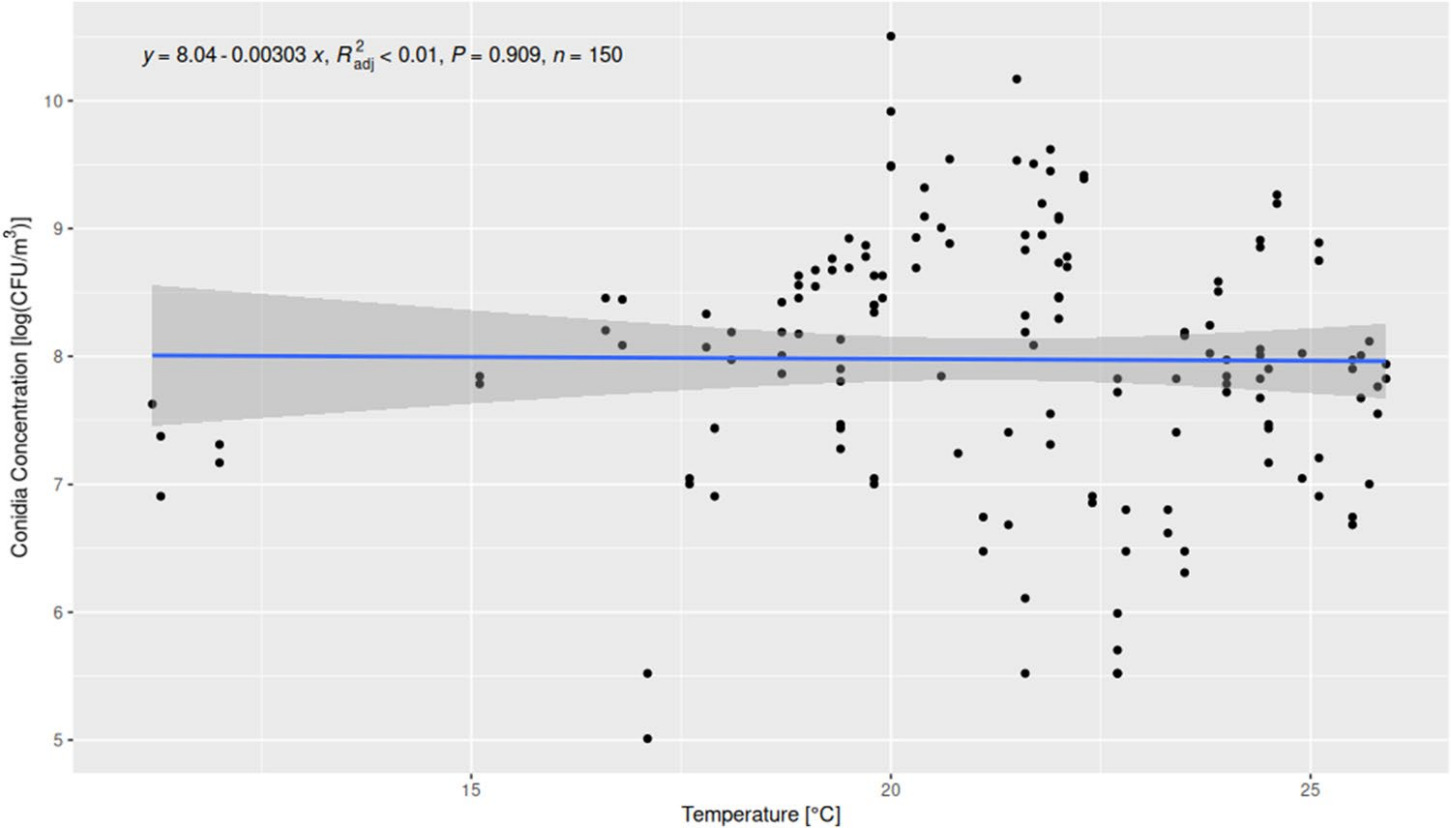
The correlation between temperature and fungal conidia concentration in the air

**Fig. 3:**
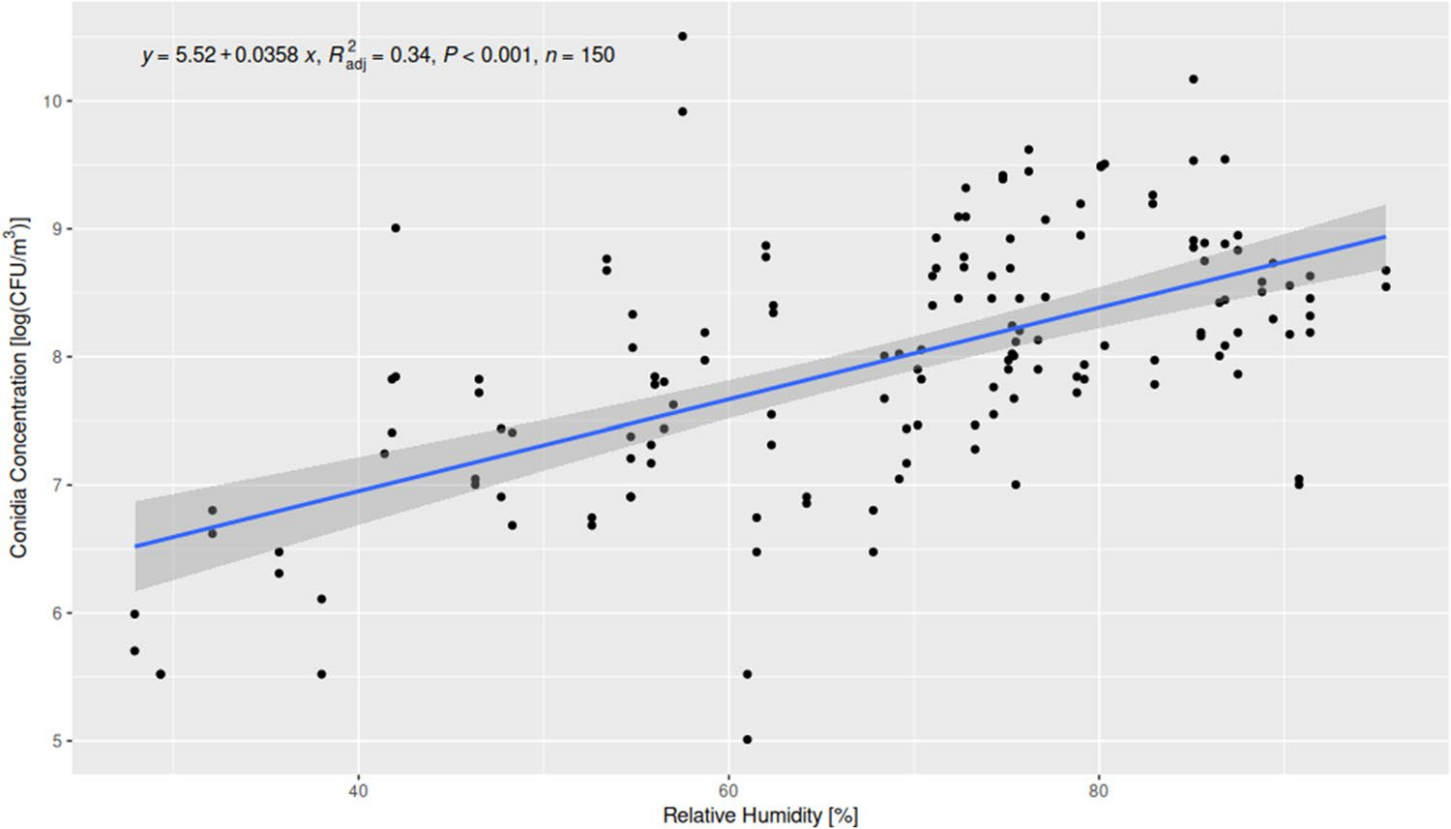
The correlation between relative humidity and fungal conidia concentration in the air

The concentration of airborne fungi varies across different seasons and is dependent on environmental factors such as temperature or relative humidity (Yuan et al. 2022). The important factors that increase the spread of fungi in the air and sustain their growth are temperature and humidity (Pflieger et al. 2020). Therefore, our studies were conducted in a seasonal cycle (spring, summer, autumn, winter), and the environmental conditions (temperature and relative air humidity) were measured during each sampling season using a thermo-hygrometer (Table 3). The lowest recorded temperature (11 °C) was noted in autumn in the room for Papio Pavilion, while the highest temperatures were noted in summer in the Kongo Pavilion (25.5–25.9 °C). Similarly, the highest humidity was observed in winter in East Africa (95.5%) and in the Kongo Pavilion. In general, the relative humidity during the studies varied from 29.3% (in the Apes Pavilion) to 95.5% in the Kongo Pavilion. Environmental parameters (temperature and relative humidity) were correlated with the total number of CFU/m^3^ noted in all locations, based on Spearman correlation analysis. The relative humidity correlated positively with the total fungal concentration (rho = 0.57, *p* < 0.0021), while temperature showed no significant correlation with the total fungal concentration at all locations (rho =  − 0.1, *p* < 0.2263) (Figs. 2 and 3).

**Table 3:** Results of air temperature and relative humidity measurements inside 20 locations in the zoologic garden in autumn, winter, spring, and summer sampling seasons

No	Autumn		Winter		Spring		Summer	
	Temp (°C)	RH (%)	Temp (°C)	RH (%)	Temp (°C)	RH (%)	T (°C)	RH (%)
1	22.7	29.3	23.3	32.1	23.4	41.8	25.1	54.7
M2	22.7	27.9	21.6	38.0	23.5	35.7	25.5	52.6
3	12	55.8	20.6	30.8	18.1	58.7	19.5	75.2
4	11.3	54,7	17.9	52.0	16.6	75.7	19.4	76.7
5	11.2	57	24.0	30.8	17.9	47.7	19.4	73.3
6	20	57.5	16.8	86.8	19.4	56.5	22.8	67.8
7	20.8	41,4	15.1	56.0	19.8	463	22.4	64.2
8	20.6	42	19.7	62.0	21.4	48.3	21.9	62.3
9	54.8	17.8	19.3	53.4	22.7	46.5	21.1	61.5
10	11	57.7	17.1	61.0	17.6	90.8	20	80.1
11	20.4	72.8	19.1	95.5	21.9	76.2	24.4	70.4
12	20.3	71.2	18.9	90.3	21.8	79	24.4	68.4
13	19.9	74.2	18.7	87.5	22.3	74.8	24.5	70.2
14	19.8	71	18.7	86.5	22.1	72.7	24.9	69.2
15	19.8	62.4	18.9	91.4	22	72.4	24.5	69.6
16	22	89.4	21.6	91.4	23.9	88.8	25.9	79.2
17	20.7	86.8	23.5	85.5	21.6	87.5	25.8	74.3
18	21.5	85.1	24.0	83.0	24.4	85.1	25.7	75.5
19	21.7	80.3	24.0	78.8	25.1	85.7	25.6	75.4
20	22	77.1	23.8	75.3	24.6	82.9	25.5	75.1

**Fig. 4:**
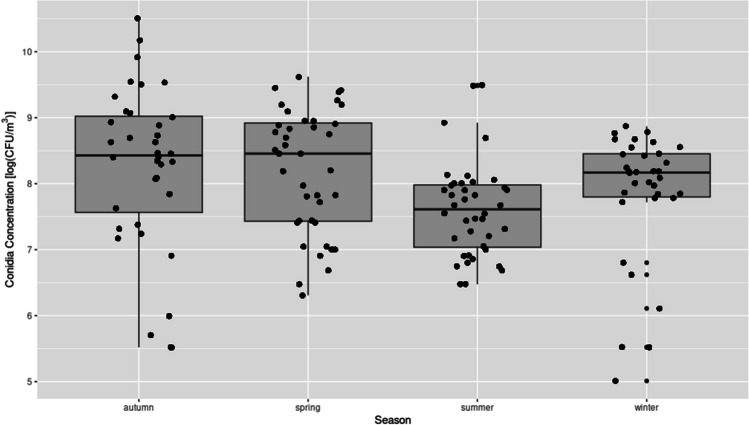
Fungal conidia concentration in the air across different seasons

The concentration of airborne fungi observed at each location varied significantly across the seasons. The highest concentration was recorded in the autumn, reaching a level of 3.65 × 10^5^ CFU/m^3^ while the lowest levels were observed in the spring and summer, with values of 1.5 × 10^4^ and 1.32 × 10^4^ CFU/m^3^, respectively. The lowest concentration of fungi (2.5 × 10^2^ CFU/m^3^) was observed in the winter season. It has been demonstrated that optimal temperature and high relative humidity can contribute to a sudden increase in the concentration of airborne fungi. The medium fungal concentrations varied considerably by season, with the greatest variation noted between summer and autumn (*p* < 0.0018). A strong variety of airborne fungal concentrations was also noticed between spring and summer (*p* < 0.0056), while no statistically significant differences were observed between other seasons (Fig. 4).

Interpretation of the results of microbiological air contamination is challenging because of the lack of acceptable limits for microbiological agents. The most commonly used measure of exposure to microbial air pollution is the degree of such pollution, expressed in terms of the number of colony-forming units (CFU) in 1 m^3^ of air (Górny et al. 2016). The threshold limit values (TLV) were used in the assessment of mycological air contamination. For microbiological agents in the air of occupational and non-occupational environments, TLV was proposed by the Expert Group on Biological Agents at the Polish Interdepartmental Commission for Maximum Admissible Concentrations and Intensities for Agents Harmful to Health in the Working Environment (Górny et al. 2016). These values (5 × 10^4^ CFU/m^3^ for fungi) were developed as a result of volumetric measurements of environmental bioaerosols. Based on the TLV proposed by the mentioned Expert Group, we can conclude that the quantitative analysis of the fungal aerosol showed lower concentration values than the recommended permissible limits. Nevertheless, this is not equivalent to the absence of microbial contamination in the facilities that were studied. Particularly as some of the values were close to TLV values. The US government agency, Occupational Safety and Health Administration (OSHA), suggests that a value higher than 1.0 × 10^3^ CFU/m^3^ indoor may be an indicator of microbial contamination. However, a determination of bioaerosol biodiversity is needed to confirm a health hazard, as certain species may pose a greater health concern than others (OSHA 2015; Rivas et al. 2018).

### Biodiversity of airborne fungi

The total number of airborne fungi varied by location (Table 4) and seasons (Table 5). The Margalef’s Index was used to analyze the biodiversity of airborne fungi throughout the study period and in different locations. The highest value of Margalef’s Index (0.93) was recorded at Kongo Pavilion in location no 18 and the lowest (0.00) was observed at East Africa Pavilion in location no 12. Concerning seasons, the highest diversity of airborne fungi was recorded in the winter (34), followed by the autumn (29), and spring (27), whereas the lowest number of strains were noted in the summer (21). The average Jaccard Index for all sites was quite low, at 0.135. The lowest average Jaccard Index, calculated pairwise for each research site, was for the Kongo Pavilion (0.12). This indicates that the Kongo Pavilion had the highest number of unique isolated species compared to other research sites. Direct comparison showed the lowest species similarity between the Kongo Pavilion and the Apes Pavilion (Jaccard Index = 0.06) and between the Kongo Pavilion and the Papio Pavilion (0.08). The highest similarity was found between the Kongo Pavilion and the East Africa Pavilion (0.22).

**Table 4:** Number of fungi species isolated from the air in the 20 locations in the zoological garden

No	Species	Zoological garden																				Total
		1	2	3	4	5	6	7	8	9	10	11	12	13	14	15	16	17	18	19	20	
1	*Aspergillus niger*		1					1								1						3
2	*Aspergillus ostianus*											1						1				2
3	*Aspergillus tubigensis*		1																			1
4	*Aspergillus elegans*	2	1												1							4
5	*Aspergillus giganteus*																			1	1	2
6	*Aspergillus ochraceus*	1	1	1	1													1				5
7	*Aspergillus steynii*																1					1
8	*Aspergillus puulaauensis*					1																1
9	*Aspergillus westerdijkiae*	1			1							1										3
10	*Aspergillus versicolor*													1								1
11	*Aspergillus fumigatus*			1	1		2															4
12	*Aspergillus flavus*		1																1			2
13	*Penicillium brevicompactum*	1	1		1					1												4
14	*Penicillium chrysogenum*												1			1					1	3
15	*Penicillium commune*					1	1								1			1				4
16	*Penicillium glabrum*	1			1			1	1	1												5
17	*Penicillium lanosocoeruleum*																	1		1		2
18	*Penicillium solitum*														1	1						2
19	*Penicillium steckii*																1		2		2	5
20	*Penicillium brasilianum*			1																		1
21	*Penicillium allii*																			2		2
22	*Penicillium citrinum*															2				1		3
23	*Penicillium bialowieziense*									1												1
24	*Penicillium copticola*																	1	2			3
25	*Penicillium griseofulvum*																	2	1	1		4
26	*Penicillium olsonii*													1								1
27	*Penicillium sumatreanse*															1	1					2
28	*Penicillium citreosulfuratum*	2																				2
29	*Penicillium hetheringtoini*																		1			1
30	*Penicillium raistrickii*									1												1
31	*Penicillium rubens*										1											1
32	*Penicillium glandicola*						1		1													2
33	*Penicillium sarajorii*								1													1
34	*Penicillium griseoroseum*							1														1
35	*Penicillium toxicarium*						1															1
36	*Penicillium vanlyukii*															1						1
37	*Penicillium* spp.						1		2		2				1	1		1	1	3	1	13
38	*Talaromyces aspriconidius*		1																			1
39	*Talaromyces minioluteus*															1						1
40	*Talaromyces divercus*										1											1
41	*Talaromyces piceae*																		1			1
42	*Cladosporium cladosporoides*			1	1																	2
43	*Cladosporium halotolerans*																		1			1
44	*Cladosporium* spp.			1						1												2
45	*Syncephalastrum racemosum*	1																				1
46	*Mucor plumbeus*						2															2
47	*Absidia* spp.		1																			1
48	*Schizophyllumcommune*				1	1																2
49	*Cuninghamella clavata*																			1		1
50	*Alternaria alternata*		1																			1
Total		9	9	5	7	3	8	3	5	5	4	2	1	2	4	9	3	8	10	10	5	**112**

1–2 (Monkey House), 3–5 (cages with maggots 1), 6–9 (Apes Pavilion), 10 (Papio Pavilion), 11–15 (Kongo Pavilion), 16–20 (East Africa pavilion)

**Table 5:** Number of fungi species isolated from the air in the seasons

No	Species	Zoological Garden				Total
		Autumn	Winter	Spring	Summer	
1	*Aspergillus niger*	3				3
2	*Aspergillus ostianus*	1			1	2
3	*Aspergillus tubigensis*	1				1
4	*Aspergillus elegans*		2		2	4
5	*Aspergillus giganteus*		2			2
6	*Aspergillus ochraceus*		2	2	1	5
7	*Aspergillus steynii*			1		1
8	*Aspergillus puulaauensis*				1	1
9	*Aspergillus westerdijkiae*		2		1	3
10	*Aspergillus versicolor*		1			1
11	*Aspergillus fumigatus*			2	2	4
12	*Aspergillus flavus*			2		2
13	*Penicillium brevicompactum*	4				4
14	*Penicillium chrysogenum*		1	1	1	3
15	*Penicillium commune*	1	2	1		4
16	*Penicillium glabrum*	1		4		5
17	*Penicillium lanosocoeruleum*	2				2
18	*Penicillium solitum*	2				2
19	*Penicillium steckii*	2	2	1		5
20	*Penicillium brasilianum*		1			1
21	*Penicillium allii*		1	1		2
22	*Penicillium citrinum*		2		1	3
23	*Penicillium bialowieziense*	1				1
24	*Penicillium copticola*		2		1	3
25	*Penicillium griseofulvum*		4			4
26	*Penicillium olsonii*		1			1
27	*Penicillium sumatreanse*		1	1		2
28	*Penicillium citreosulfuratum*		2			2
29	*Penicillium hetheringtoini*		1			1
30	*Penicillium raistrickii*			1		1
31	*Penicillium rubens*		1			1
32	*Penicillium glandicola*			2		2
33	*Penicillium sarajorii*			1		1
34	*Penicillium griseoroseum*				1	1
35	*Penicillium toxicarium*				1	1
36	*Penicillium vanlyukii*				1	1
37	*Penicillium* spp.	7	3	2	1	13
38	*Talaromyces aspriconidius*			1		1
39	*Talaromyces minioluteus*				1	1
40	*Talaromyces divercus*				1	1
41	*Talaromyces piceae*		1			1
42	*Cladosporium cladosporoides*	2				2
43	*Cladosporium halotolerans*	1				1
44	*Cladosporium* sp.	1		1		2
45	*Syncephalastrum racemosum*			1		1
46	*Mucor plumbeus*			1	1	2
47	*Absidia* spp.			1		1
48	*Schizophyllum commune*				2	2
49	*Cuninghamella clavata*				1	1
50	*Alternaria alternata*					1
Total		29	34	27	21	**112**

We used both morphological and molecular identity methods based on sequencing regions of the ITS, b-tubulin, and calmodulin. Using these methods, a total of 112 fungal strains belonging to 10 genera and 50 species were isolated during the whole 1-year-long study. The molecular identification corresponded well with the morphological diagnosis and proved to be a good tool for diagnostic strains that were not identified microscopically. By comparing the partial sequences of the ITS region of the own isolates with the sequences of other isolates available in GenBank, the results showed that the similarity percentage of nucleotide sequences ranged between 95 and 100% (Supplement 1). In most cases, (87%) the sequence data allowed the identification at the species level. The study based on the molecular analysis allowed us to identify 97 out of 112 species of fungi. Diagnosis of thirteen strains belonged to *Penicillium* genera, and one strain each of the *Cladosporium* and *Absidia* genera failed. Analysis of ITS region revealed that they belong to closely related species. Even the sequencing of the less conservative region (b-tubulin) and calmodulin did not allow for the identification of these fungi at the species level.

**Fig. 5:**
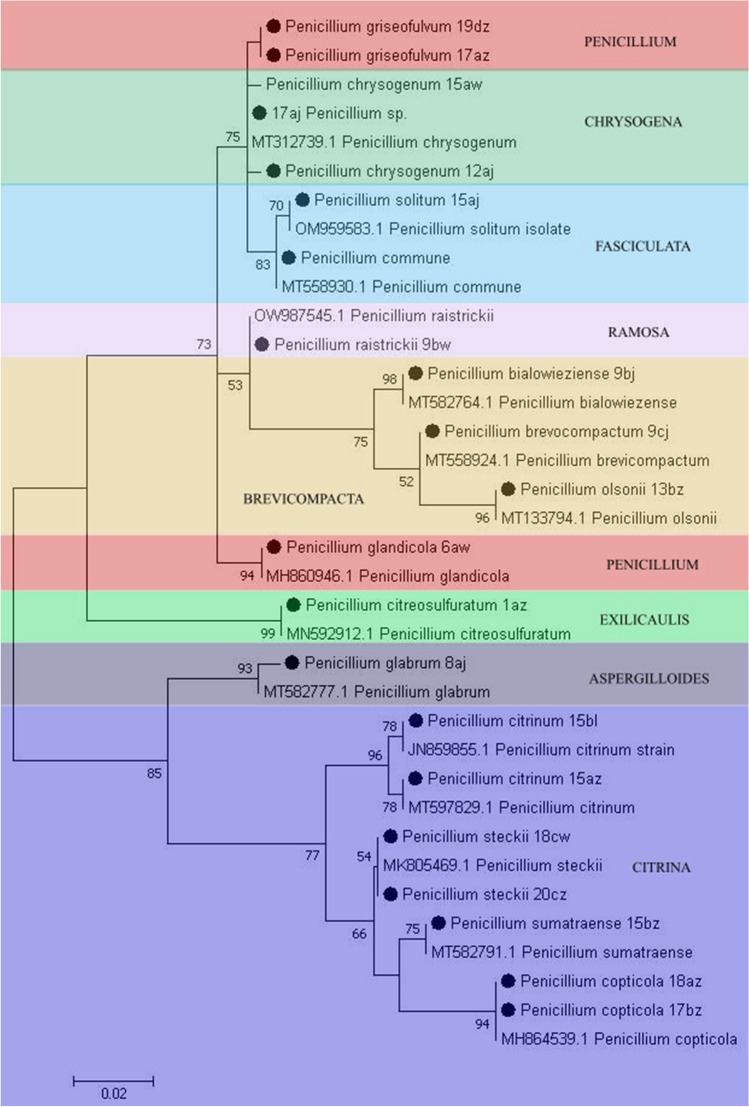
Maximum likelihood (ML) phylogenetic tree (*Tamura 3*-*parameter* + *G*) for *Penicillium sp.* based on sequences of the ITS gene fragment. Bootstrap values are shown above the branches. Sequences from this study are marked with solid circles. The dendrogram was constructed with 1000 replications using MEGA software

**Fig. 6:**
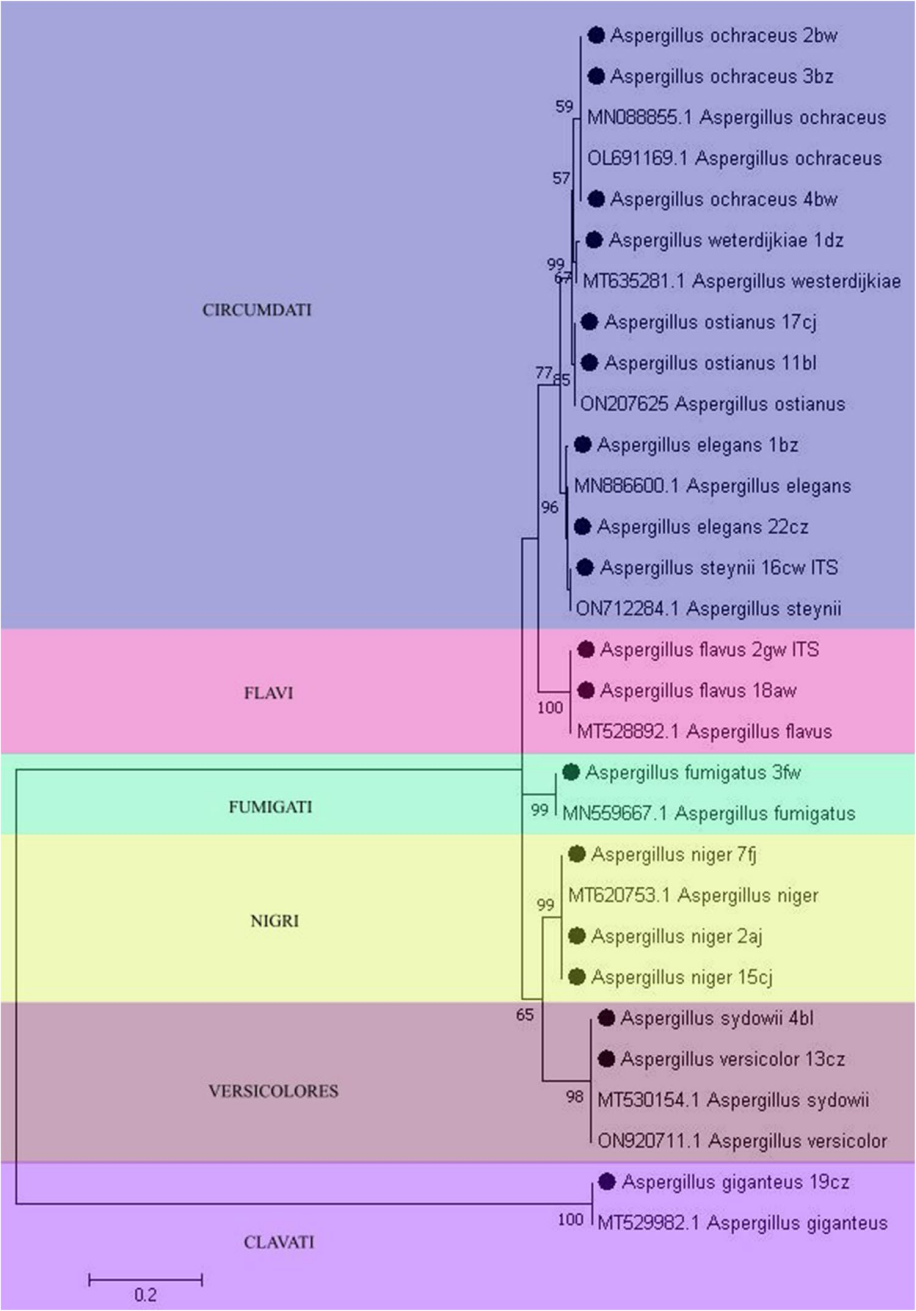
Maximum likelihood (ML) phylogenetic tree (*Jukes-Cantor* + *G*) for *Aspergillus* sp. based on sequences of the ITS gene fragment. Bootstrap values are shown above the branches. Sequences from this study are marked with solid circles. The dendrogram was constructed with 1000 replications using MEGA software

Most of the airborne fungi isolated in our studies were representatives of Ascomycota (93.75%), while Mucormycota constituted 4.46% of the mycobiota and Basidiomycota for 1.79%. *Penicillium* was the dominant genera, including 58.9% of total fungal strains, followed by *Aspergillus* 25.89%, *Cladosporium* 3.57%, *Talaromyces* 3.57%, *Mucor* 1.78%, *Schizophyllum* 1.78%, *Syncephalastrum* 0.89%, *Alternaria* 0.89%, *Absidia* 0.89%, and *Cunninghamella* 0.89%. Species belonging to the most representative *Penicillium* genera were grouped in the seven following sections such as the following: Fasciculata (*P. solitum*, *P. commune*, *P. allii*), Chrysogena (*P. chrysogenum*, *P. lanosocoeruleum*, *P. rubens*, *P. vanlyukii*), Penicillium (*P. griseofulvum*, *P. glandicola*), Ramosa (*P. rastrickii*), Brevicompacta (*P. olsonii*, *P. brevicompactum*, *P. bialowieziense*), Exilicaulis (*P. citreosulfuratum*), Aspergilloides (*P. glabrum*), Citrina (*P. citrinum*, *P. copticola*, *P. sumataense*, *P. steckii*, *P. hetheringtonii*) (Fig. 5). *Aspergillus* genera were represented by six dominant sections: Nigri (*A. niger*, *A. tubigensis*), Circumdati (*A. ochraceus*, *A. westerdijkiae*, *A. ostianus*, *A. elegans*, *A. steynii*), Flavi (*A. flavus*), Fumigatus (*A. fumigatus*), Versicolores (*A. versicolor*, *A. sydowii*, *A. puulaauensis*) (Fig. 6).

Diversity of identified fungi varied between different locations and seasons. Unfortunately, there is a lack of research about the biodiversity of airborne fungi in zoological gardens. Therefore, our results could be compared to the studies obtained for the livestock premises. For instance, in the China poultry houses, researchers observed a different genus composition than in our studies. They isolated mainly *Trichosporon*, *Candida*, *Aspergillus*, *Cladosporium*, and *Alternaria* genera (Chen et al. 2021). In other studies, *Aspergillus*, *Scopulariopsis*, *Penicillium*, and *Cladosporium* were detected in the air of the swine house (Kumari et al. 2016). Comparable results to ours have been reported by Matković et al. 2009. Croatian authors detected *Aspergillus* and *Penicillium* genera in dwellings for dairy cows and laying hens.

Some of the fungi isolated in our research may be associated with allergic respiratory diseases (especially in people with a weakened immune system). Filamentous fungi can be a source of polysaccharides such as the β(1 → 3)-glucans. This compound may cause inflammatory airway reactions and also affect the immune system. There is increasing evidence that β(1 → 3)-glucans cause non-specific inflammatory reactions and can be responsible for bioaerosol-induced respiratory symptoms observed in both indoor and occupational environments (Pflieger et al. 2020). The other fungi are important producers of mycotoxins, which have been suggested as one of the major possible causes of health problems. For example, several detected fungi belonging to *Aspergilli* in sections *Circumdati* (*A. steynii* and *A. westerdijkiae*), *Flavi* (*A. flavus*), and *Nigri* (*A. carbonarius* and *A. niger*) are well-known producers of ochratoxins, a mycotoxin teratogenic, carcinogenic, immunosuppressive, and nephrotoxic in animals (Kagot et al. 2019). The other species, such as *A. versicolor* and *A. flavus*, produce sterigmatocystin and aflatoxins, respectively. Aflatoxins are the most potent natural carcinogens (Lamoth 2016). What’s more, the identified in our study *A. flavus* is recognized as an opportunistic animal and human pathogen, while *A.* *fumigatus* is the main causative agent of pulmonary invasive aspergillosis (Al-Shaarani et al. 2023). The most abundant *Penicillium* genera isolated in our studies is listed among the most common allergenic fungal taxa and has been linked to asthma. Whereas, *Schizophyllum commune*, which accounted for 1.78%, may be the cause of ABPM (allergic bronchopulmonary mycosis) and allergy-related bronchopulmonary infections and sinusitis (Oguma et al. 2023).

## Conclusion

This is the first research on culturable airborne fungi at the zoological garden in Poland. The quantitative analysis of the fungal aerosol showed that the obtained concentration values were lower than the recommended permissible limits. But a lot of detected fungi can be harmful to human and animal health. That is why, our study emphasizes the necessity of air quality monitoring.

Similar studies to ours are rarely conducted. Therefore, our preliminary research provides basic information about the fungal concentrations and their biodiversity in these touristic facilities. However, further long-term quantitative, as well as qualitative, and mycotoxicological research is needed to fully understand airborne fungal composition in the zoological garden and its potentially negative impact on human and animal health. Moreover, quantitative and qualitative assessment of mycotoxins in the air is of great importance from the occupational exposure point of view. In addition, our preliminary results along with planned long-term studies of mycological air contamination in the zoo may contribute in the future to the development of microbiological air quality standards by relevant institutions.

### Supplementary Information

Below is the link to the electronic supplementary material.

Supplementary file1 (DOCX 30 KB)

## Data Availability

All data generated or analyzed during this study are included in this published article.
