# Supplementary material for: Mycological air contamination level and biodiversity of airborne fungi isolated from the zoological garden air — preliminary research
Source: Environ Sci Pollut Res Int. 2024 Jun 18;31(30):43066–79. doi: 10.1007/s11356-024-33926-2 (PMC11222260; doi:10.1007/s11356-024-33926-2)
Supplement: Supplementary file 1 — Supplementary file1 (DOCX 30 KB) [file 11356_2024_33926_MOESM1_ESM.docx]

| Supplement 1. Molecular identification (using ITS region) of fungi isolated from the air in the zoological garden. | | | | | |
| --- | --- | --- | --- | --- | --- |
|  |  |  |  | **Identity with sequence from GenBank** | |
| **Fungal species** | **Isolate** | **Location** | **Season** | **Accesion No.** | **Identity** |
| *Aspergillus niger (1)* | 7fj | 1 | Autumn | [MT620753.1](https://www.ncbi.nlm.nih.gov/nucleotide/MT620753.1?report=genbank&log$=nucltop&blast_rank=1&RID=H7UFZ30G013) | 100% |
| *Aspergillus niger (2)* | 15cj | 15 | Autumn | [MT620753.1](https://www.ncbi.nlm.nih.gov/nucleotide/MT620753.1?report=genbank&log$=nucltop&blast_rank=1&RID=H7V6BCMY013) | 100% |
| *Aspergillus niger (3)* | 2aj | 2 | Autumn | [MT620753.1](https://www.ncbi.nlm.nih.gov/nucleotide/MT620753.1?report=genbank&log$=nucltop&blast_rank=1&RID=H7VEY6JY013) | 100% |
| *Aspergillus ostianus* | 17cj | 17 | Autumn | [ON207625.1](https://www.ncbi.nlm.nih.gov/nucleotide/ON207625.1?report=genbank&log$=nucltop&blast_rank=1&RID=H7VJB3UJ016) | 100% |
| *Cladosporium cladosporoides (1)* | 3j | 3 | Autumn | MT573533.1 | 100% |
| *Cladosporium cladosporoides (2)* | 4ej | 4 | Autumn | [MT635286.1](https://www.ncbi.nlm.nih.gov/nucleotide/MT635286.1?report=genbank&log$=nucltop&blast_rank=1&RID=H7W2HD6Y016) | 100% |
| *Cladosporium halotolerans* | 17bj | 17 | Autumn | [MT635287.1](https://www.ncbi.nlm.nih.gov/nucleotide/MT635287.1?report=genbank&log$=nucltop&blast_rank=2&RID=H7W4ZBDP016) | 100% |
| *Cladosporium* spp. *(1)* | 9dj | 9 | Autumn | [MT635286.1](https://www.ncbi.nlm.nih.gov/nucleotide/MT635286.1?report=genbank&log$=nucltop&blast_rank=1&RID=H7W7575H016) | 100% |
| *Penicillium bialowieziense* | 9bj | 9 | Autumn | [MT582764.1](https://www.ncbi.nlm.nih.gov/nucleotide/MT582764.1?report=genbank&log$=nucltop&blast_rank=1&RID=H7WJEP9W016) | 100% |
| *Penicillium brevicompactum (1)* | 9cj | 9 | Autumn | [MT558924.1](https://www.ncbi.nlm.nih.gov/nucleotide/MT558924.1?report=genbank&log$=nucltop&blast_rank=1&RID=H7WUFEJS016) | 100% |
| *Penicillium brevicompactum (2)* | 1ej | 1 | Autumn | [MN105179.1](https://www.ncbi.nlm.nih.gov/nucleotide/MN105179.1?report=genbank&log$=nucltop&blast_rank=1&RID=H7WX8X1Y013) | 100% |
| *Penicillium brevicompactum (3)* | 4aj | 4 | Autumn | [MT582765.1](https://www.ncbi.nlm.nih.gov/nucleotide/MT582765.1?report=genbank&log$=nucltop&blast_rank=1&RID=H7ZEBZ10013) | 100% |
| *Penicillium brevicompactum (4)* | 2bj | 2 | Autumn | [MT558924.1](https://www.ncbi.nlm.nih.gov/nucleotide/MT558924.1?report=genbank&log$=nucltop&blast_rank=1&RID=H7WZBND3013) | 100% |
| *Penicillium chrysogenum* | 12aj | 12 | Autumn | [MK696221.1](https://www.ncbi.nlm.nih.gov/nucleotide/MK696221.1?report=genbank&log$=nucltop&blast_rank=1&RID=H7X2G9HM013) | 99,82% |
| *Penicillium commune* | 6dj | 6 | Autumn | [MT558930.1](https://www.ncbi.nlm.nih.gov/nucleotide/MT558930.1?report=genbank&log$=nucltop&blast_rank=2&RID=H7X87TD2016) | 100% |
| *Penicillium glabrum* | 8aj | 8 | Autumn | [MT582777.1](https://www.ncbi.nlm.nih.gov/nucleotide/MT582777.1?report=genbank&log$=nucltop&blast_rank=1&RID=H7XDGMJY013) | 100% |
| *Penicillium* spp | 17aj | 17 | Autumn | [MH856372.1](https://www.ncbi.nlm.nih.gov/nucleotide/MH856372.1?report=genbank&log$=nucltop&blast_rank=2&RID=H7XGERPZ013) | 99,82% |
| *Penicillium lanosocoeruleum (2)* | 19aj | 19 | Autumn | [MH856372.1](https://www.ncbi.nlm.nih.gov/nucleotide/MH856372.1?report=genbank&log$=nucltop&blast_rank=2&RID=H7YBSGPU01R) | 100% |
| *Penicillium solitum (1)* | 14ej | 14 | Autumn | [MK682879.1](https://www.ncbi.nlm.nih.gov/nucleotide/MK682879.1?report=genbank&log$=nucltop&blast_rank=1&RID=H7XNS3DN013) | 100% |
| *Penicillium solitum(2)* | 15aj | 15 | Autumn | [OM959583.1](https://www.ncbi.nlm.nih.gov/nucleotide/OM959583.1?report=genbank&log$=nucltop&blast_rank=1&RID=H7XT766J013) | 99,63% |
| *Penicillium steckii (1)* | 16bj | 16 | Autumn | [MN187973.1](https://www.ncbi.nlm.nih.gov/nucleotide/MN187973.1?report=genbank&log$=nucltop&blast_rank=3&RID=H7ZGYJNX016) | 99,82% |
|  |  |  |  |  |  |
| *Aspergillus elegans (1)* | 1bz | 1 | Winter | [MN886600.1](https://www.ncbi.nlm.nih.gov/nucleotide/MN886600.1?report=genbank&log$=nucltop&blast_rank=2&RID=HA7MPH0G016) | 99% |
| *Aspergillus elegans (2)* | 2cz | 2 | Winter | [MN886600.1](https://www.ncbi.nlm.nih.gov/nucleotide/MN886600.1?report=genbank&log$=nucltop&blast_rank=1&RID=HA7UVE7G016) | 100% |
| *Aspergillus giganteus (1)* | 19cz | 19 | Winter | MT529982.1 | 100% |
| *Aspergillus giganteus (2)* | 20dz | 20 | Winter | MT529982.1 | 100% |
| *Aspergillus ochraceus (1)* | 17ez | 17 | Winter | MN088855.1 | 100% |
| *Aspergillus ochraceus (2)* | 3bz | 3 | Winter | [OL691169.1](https://www.ncbi.nlm.nih.gov/nucleotide/OL691169.1?report=genbank&log$=nucltop&blast_rank=1&RID=HA8R4KRG016) | 100% |
| *Aspergillus versicolor* | 13cz | 13 | Winter | ON920711.1 | 99% |
| *Aspergillus weterdijkiae (1)* | 1dz | 1 | Winter | MT635281.1 | 99.55% |
| *Aspergillus weterdijkiae (2)* | 4dz | 4 | Winter | MT635281.2 | 99.55% |
| *Penicillium allii* | 19ez | 19 | Winter | AY678584.1 | 99% |
| *Penicillium brasilianum* | 3cz | 3 | Winter | MN401031.1 | 100% |
| *Penicillium chrysogenum* | 20aez | 20 | Winter | MT229079.1 | 100% |
| *Penicillium citrinum (1)* | 15az | 15 | Winter | MT597829.1 | 100% |
| *Penicllium citrinum (2)* | 19bz | 19 | Winter | JN859855.1 | 100,00% |
| *Penicillium commune (1)* | 14bz | 14 | Winter | KT316690.1 | 98.94 |
| *Penicillium commune (2)* | 17fz | 17 | Winter | KY606533.1 | 99% |
| *Penicillium copticola (1)* | 17bz | 17 | Winter | MH864539.1 | 100,00% |
| *Penicillium copticola (2)* | 18az | 18 | Winter | MH864539.1 | 100% |
| *Penicillium griseofulvum(1)* | 17az | 17 | Winter | [MT378399.1](https://www.ncbi.nlm.nih.gov/nucleotide/MT378399.1?report=genbank&log$=nucltop&blast_rank=1&RID=HAAWT5NA016) | 100% |
| *Penicillium griseofulvum (2)* | 17cz | 17 | Winter | [MT378399.1](https://www.ncbi.nlm.nih.gov/nucleotide/MT378399.1?report=genbank&log$=nucltop&blast_rank=1&RID=HAAWT5NA016) | 100% |
| *Penicillium griseofulvum(3)* | 18cz | 18 | Winter | [MT378399.1](https://www.ncbi.nlm.nih.gov/nucleotide/MT378399.1?report=genbank&log$=nucltop&blast_rank=1&RID=HAAWT5NA016) | 100% |
| *Penicillium griseofulvum(3)* | 19dz | 19 | Winter | [MT378399.1](https://www.ncbi.nlm.nih.gov/nucleotide/MT378399.1?report=genbank&log$=nucltop&blast_rank=1&RID=HAAWT5NA016) | 100% |
| *Penicillium olsonii* | 13bz | 13 | Winter | MT133794.1 | 100% |
| *Penicillium steckii (1)* | 18bz | 18 | Winter | MK805469.1 | 100% |
| *Penicillium steckii (2)* | 20cz | 20 | Winter | MK805469.1 | 100% |
| *Penicillium sumatraense* | 15bz | 15 | Winter | MT582791.1 | 100,00% |
| *Penicillium citreosulfuratum* | 1az | 1 | Winter | MN592912.1 | 100% |
| *Penicillium* spp. *(1)* | 15bz | 15 | Winter | MT582791.1 | 100% |
| *Talaromyces piceae* | 16bz | 16 | Winter | [KF984784.1](https://www.ncbi.nlm.nih.gov/nucleotide/KF984784.1?report=genbank&log$=nucltop&blast_rank=3&RID=HAD2CF4H013) | 100 |
|  |  |  |  |  |  |
| *Aspergillus flavus (1)* | 2gw | 2 | Spring | OR397999.1 | 100% |
| *Aspergillus flavus (2)* | 18aw | 18 | Spring | MT528892.1 | 100% |
| *Aspergillus fumigatus (1)* | 3fw | 3 | Spring | MN559667.1 | 100% |
| *Aspergillus fumigatus (2)* | 4gw | 4 | Spring | MN559667.1 | 100% |
| *Aspergillus ochraceus (1)* | 2bw | 2 | Spring | MN088855.1 | 100% |
| *Aspergillus ochraceus (2)* | 4bw | 4 | Spring | MT497402.1 | 100% |
| *Aspergillus steynii* | 16cw | 16 | Spring | ON712284.1 | 100% |
| *Penicillium chrysogenum* | 15aw | 15 | Spring | JQ082504.1 | 100% |
| *Penicillium glabrum (1)* | 1bw | 1 | Spring | MN856208.1 | 100% |
| *Penicillium glabrum (2)* | 4cw | 4 | Spring | MT441616.1 | 100% |
| *Penicillium raistrickii* | 9bw | 9 | Spring | OW987545.1 | 100% |
| *Penicillium sumatreansae* | 16dw | 16 | Spring | MK910053.1 | 99,15% |
| *Penicillium* spp. *(1)* | 6bw | 6 | Spring | KY921948.1 | 99% |
| *Penicillium* spp. *(2)* | 8bw | 8 | Spring | MT441635.1 | 100% |
| *Penicillium commune* | 5aw | 5 | Spring | KY606533.1 | 99% |
| *Penicilllium steckii* | 18cw | 3 | Spring | MK805469.1 | 100% |
| *Penicillium glandicola* | 6aw | 6 | Spring | MH860946.1 | 100,00% |
| *Syncephalastrum racemosum* | 1dw | 1 | Spring | KP067274.1 | 100,00% |
| *Mucor plumbeus* | 6cw | 6 | Spring | MK268150.1 | 100% |
| *Absidia* spp. | 2fw | 2 | Spring | OR205235.1 | 95% |
| *Cladosporium* spp. | 3aw | 3 | Spring | OQ186134.1 | 100% |
|  |  |  |  |  |  |
| *Aspergillus ostianus* | 11bl | 11 | Summer | MK805448.1 | 100% |
| *Aspergillus ochraceus* | 1bl | 1 | Summer | MK252003.1 | 100% |
| *Aspergillus westerdijikiae* | 11a | 11 | Summer | MT635281.1 | 99.55% |
| *Aspergillus fumigatus* | 6d | 6 | Summer | [MT420414.1](https://www.ncbi.nlm.nih.gov/nucleotide/MT420414.1?report=genbank&log$=nucltop&blast_rank=1&RID=HBJNA1M8013) | 100% |
| *Aspergillus fumigatus* | 6c | 6 | Summer | [MT420414.1](https://www.ncbi.nlm.nih.gov/nucleotide/MT420414.1?report=genbank&log$=nucltop&blast_rank=1&RID=HBJT1EG3016) | 99.58% |
| *Aspergillus sydowii* | 4b | 4 | Summer | [MT530154.1](https://www.ncbi.nlm.nih.gov/nucleotide/MT530154.1?report=genbank&log$=nucltop&blast_rank=1&RID=HBN8BK16016) | 99.81% |
| *Aspergillus elegans* | 14al | 14 | Summer | [MN886600.1](https://www.ncbi.nlm.nih.gov/nucleotide/MN886600.1?report=genbank&log$=nucltop&blast_rank=1&RID=HA7UVE7G016) | 100% |
| *Talaromyces minioluteus* | 15al | 15 | Summer | MN311451.1 | 100% |
| *Mucor plumbeus* | 6al | 6 | Summer | MK268150.1 | 100% |
| *Alternaria alternata* | 2c | 2 | Summer | [MT524319.1](https://www.ncbi.nlm.nih.gov/nucleotide/MT524319.1?report=genbank&log$=nucltop&blast_rank=1&RID=HBJ9JREX013) | 100% |
| [Schizophyllum commune](https://blast.ncbi.nlm.nih.gov/Blast.cgi#alnHdr_1786849124) | 5al | 5 | Summer | MN856414.1 | 100% |
| *Schizophyllum commune* | 4a | 4 | Summer | [MK934578.1](https://www.ncbi.nlm.nih.gov/nucleotide/MK934578.1?report=genbank&log$=nucltop&blast_rank=1&RID=HBNJR620016) | 100,00% |
| *Cuninghamella clavata* | 19bj | 19 | Summer | [JN205890.1](https://www.ncbi.nlm.nih.gov/nucleotide/JN205890.1?report=genbank&log$=nucltop&blast_rank=1&RID=HBP19MZ9013) | 95% |
| *Penicillium vanlyukii* | 15bl | 15 | Summer | [MK451671.1](https://www.ncbi.nlm.nih.gov/nucleotide/MK451671.1?report=genbank&log$=nucltop&blast_rank=1&RID=HBDXHFBB013) | 99% |
| *Penicillium* spp. | 8dl | 8 | Summer | MT582791.1 | 100% |
| *Penicillium citrinum* | 15dl | 15 | Summer | JN859855.1 | 100,00% |
